# Caprine herpes virus-1 reduces cell viability and enhances chemosensitivity in breast cancer cells

**DOI:** 10.3389/fonc.2025.1676296

**Published:** 2025-11-24

**Authors:** C. A. Iannuzzi, F. Pagano, M. Tomeo, A. Sfera, A. Calabrese, L. Alfano, R. Carmerlingo, S. Cocco, S. Damiano, Serena Montagnaro, R. Ciarcia, A. Giordano, M. De Laurentiis, C. von Arx, I. M. Forte

**Affiliations:** 1 Experimental Clinical Oncology of Breast Unit, Department of Breast and Thoracic Oncology, Istituto Nazionale Tumor IRCCS Fondazione G. Pascale (IRCCS) “Fondazione G. Pascale”, Naples, Italy; 2 Department of Mental and Physical Health and Preventive Medicine, Università degli Studi della Campania “Luigi Vanvitelli”, Naples, Italy; 3 Clinical and Translational Oncology Program, Scuola Superiore Meridionale (SSM), School of Advanced Studies), University of Naples Federico II, Naples, Italy; 4 Department of Medical Biotechnologies, University of Siena, Siena, Italy; 5 Cell Biology and Biotherapy Unit, Istituto Nazionale Tumori, IRCCS, Fondazione G. Pascale, Naples, Italy; 6 Department of Veterinary Medicine and Animal Productions, University of Naples “Federico II”, Napoli, Italy; 7 Sbarro Institute for Cancer Research and Molecular Medicine, Center for Biotechnology, Department of Biology, College of Science and Technology, Temple University, Philadelphia, PA, United States

**Keywords:** oncolytic virus, CpHV-1, breast cancer, targeted therapy, viral oncology, combination therapy

## Abstract

**Introduction:**

Oncolytic viruses (OVs) are promising therapeutic agents in oncology that directly lyse tumor cells, modulate the immune response, and alter the tumor microenvironment. Non-human OVs offer advantages over their human counterparts, such as being non-pathogenic in humans and lacking pre-existing immunity. In previous studies, we demonstrated that a non-human caprine herpesvirus 1 (CpHV-1) effectively kills various human cancer cell lines. In this study, we evaluate CpHV-1’s antitumor effects across different breast cancer (BC) cell lines and its potential synergy with FDA-approved BC therapies.

**Methods:**

We assessed the effects of CpHV-1 on BC cell viability and clonogenic potential, cell cycle regulation, and apoptosis in MCF-7, T47D, SKBR3, and MDA-MB-468 cell lines, as well as in non-tumorigenic mammary epithelial cells (MCF-10A). Additionally, CpHV-1 was tested in combination with Abemaciclib, Tucatinib, and Inavolisib, and synergism was evaluated using Chou–Talalay analysis.

**Results:**

Our data show that CpHV-1 induced a dose-dependent cytotoxic effect, with an MOI of 5 reducing viability by ~50% 72 hours post-infection. Clonogenic assays confirmed long-term growth inhibition. We also demonstrated modulation of cell cycle progression and induction of apoptosis in tumor cells mediated by CpHV-1. Finally, combined treatments showed synergy across all BC subtypes, without significant toxicity in normal cells.

**Discussion:**

These findings highlight CpHV-1 as a promising oncolytic agent capable of targeting multiple breast cancer subtypes. Its ability to significantly reduce viability, impair long-term proliferation, and induce apoptosis, together with its synergistic activity when combined with FDA-approved targeted therapies and its limited toxicity in normal cells, supports further investigation of CpHV-1 for breast cancer treatment.

## 1 Introduction

Breast cancer (BC) is a heterogeneous malignancy originating from the epithelial cells of the breast ducts or lobules (1, 2). Its etiology is multifactorial, involving a combination of genetic, hormonal, and environmental factors, including age, family history, BRCA1/2 mutations, hormonal influences, alcohol consumption, and obesity (3). Histologically, BC comprises invasive ductal carcinoma (IDC, ~70–80% of cases), invasive lobular carcinoma (ILC, ~10–15%), and less frequent special types (4–6). This morphological and molecular heterogeneity translates into varying tumor aggressiveness, prognosis, and therapeutic response (2). From a molecular perspective, histological classification based on estrogen (ER) and progesterone receptor (PgR) expression, HER2 amplification, and the Ki67 proliferation index identifies four intrinsic subtypes: Luminal A, Luminal B, HER2-positive (HER2+), and Triple-Negative (TNBC) (1, 2, 4, 5). Luminal tumors typically respond to endocrine therapies (anti-estrogens), often combined with CDK4/6 inhibitors (e.g., abemaciclib), PI3K inhibitors (inavolisib), or mTOR inhibitors (1, 4, 7). HER2+ tumors benefit from targeted anti-HER2 strategies, including monoclonal antibodies, tyrosine kinase inhibitors (e.g., tucatinib), and antibody–drug conjugates (ADCs) (1, 4, 8). TNBC, lacking molecular targets, has historically been treated with chemotherapy, but recent years have introduced immune checkpoint inhibitors, novel ADCs, and combination strategies including PI3K inhibitors (1, 4, 9). Despite these advances, BC remains the second leading cause of cancer-related mortality in women worldwide, with treatment-related toxicities further impacting quality of life (6, 10, 11). This underscores the need for innovative and less toxic therapies to improve outcomes.

Oncolytic virotherapy (OVT) represents an emerging anticancer modality based on genetically engineered or naturally occurring viruses that selectively infect and lyse tumor cells while sparing normal tissues (12, 13). Beyond direct cytolysis, OVs can convert immunologically “cold” tumors, such as BC, into “hot” tumors by stimulating immune infiltration and antitumor immunity (4, 5). This dual action enhances the efficacy of immunotherapies, as demonstrated in preclinical and early clinical studies (5, 12). For instance, pelareorep (Reolysin) combined with paclitaxel improved progression-free survival and immune activation in metastatic BC, whereas monotherapy showed limited benefit (1, 6, 14). Talimogene laherparepvec (T-VEC), the most advanced OV, demonstrated promising pathologic complete response rates in a phase II TNBC trial when combined with neoadjuvant chemotherapy (15). Similarly, liposomal adenoviral vectors (Ad-hTERT) are under investigation in TNBC, showing enhanced delivery, T-cell infiltration, and reduced metastasis in preclinical models (11). Despite these encouraging results, no OV has yet received regulatory approval for BC treatment (16, 17).

Among the various candidates, non-human wild-type OVs offer distinct advantages over human-derived viruses, including the inability to replicate in healthy human tissues and the absence of pre-existing immunity, while retaining natural tropism for malignant cells (14, 18). Caprine herpesvirus 1 (CpHV-1), a DNA virus, a goat pathogen related to bovine alphaherpesvirus 1, is non-pathogenic in humans (19, 20). Notably, CpHV-1 efficiently replicates in multiple human cancer cell lines, including the aggressive TNBC MDA-MB-468 model, inducing cell death via caspase-dependent apoptosis and autophagy (7, 17, 18, 21). This dual mechanism of cell death may help overcome resistance pathways that limit the efficacy of other OVs.

In this study, we assessed the oncolytic potential of CpHV-1 across a panel of molecularly distinct BC cell lines, both as monotherapy and in combination with targeted therapies. Specifically, we tested CpHV-1 with abemaciclib (for HR+/HER2− BC) (7), tucatinib (for advanced HER2+ BC) (8), and inavolisib (under clinical evaluation for TNBC) (9). The rationale for these combinations lies in the synergistic potential of OVs with subtype-specific targeted agents, leveraging CpHV-1’s cytolytic and immunostimulatory properties alongside precision oncology strategies (22).

Our findings suggest that CpHV-1, alone and in rational combinations, represents a promising new therapeutic candidate for BC. This study provides critical preclinical proof-of-concept supporting the translational development of CpHV-1-based regimens as a novel and potentially less toxic therapeutic approach to overcome resistance and improve clinical outcomes in heterogeneous BC subtypes.

## 2 Materials and methods

### 2.1 Cell lines and culture conditions

MCF-7, T47D, SKBR3 and MDA-MB-468 BC cell lines and MCF-10A non-tumorigenic mammary epithelial cells were purchased from American Type Culture Collection (ATCC; Manassas, VA, USA). MCF7, SKBR3 and MCF-10A cells were maintained in DMEM (Sigma-Aldrich, Merk KGaA, Darmstadt, Germany), MDA-MB-468 in DMEM-F12 and T47D cells in RPMI-1640 (Sigma-Aldrich, Merk KGaA, Darmstadt, Germany). All media were supplemented with 10% FBS, 10000 U/ml penicillin, 10 mg/ml streptomycin and 4 mM-glutamine (Sigma-Aldrich, Merk KGaA, Darmstadt, Germany) in a humidified atmosphere composed of 95% air and 5% CO2 at 37°C. Cell lines were regularly tested with the PlasmoTestTM Mycoplasma Detection kit (Cat. no. rep-pt1; Invivogen, San Diego, CA, USA) for the presence of mycoplasma.

### 2.2 Virus production

The reference Swiss strain E/CH19 (23)of CpHV-1 was used. It was grown on MDBK cells; viral stocks were obtained by three cycles of freezing and stored in aliquots at −80°C as previously described (24). The virus was multiplied on the MDBK cell line. Cell extracts, obtained by three cycles of freezing and thawing, were pooled, collected, and stored in aliquots at -80°C. Before use, the viral solution was partially purified by centrifugation at 3000 rpm for 20 min to remove cell debris, then pooled and stored in aliquots at -80°C (21). Infectivity titers were expressed as median tissue culture infectious doses (TCID50)/ml (25).

### 2.3 Cell infection with CpHV-1, MTS, and clonogenic assay

MCF-7, T47D, SKBR3 and MDA-MB-468 BC cell lines and MCF-10A non-tumorigenic mammary epithelial cells were seeded in triplicate in 96-well plates at a density of 1500 cells/well and allowed to adhere for 24h. Cells were then infected with CpHV-1 at doses ranging from 1.25 to 10 MOI for 72h. After treatment, cell viability was assessed using the MTS assay (Cat. no. G3582; Promega, Milan, Italy), according to the manufacturer’s instructions.

For the clonogenic assay, 500 cells were seeded in each well of 6-well plates and 24h after seeding, they were treated with CpHV-1 for 72h at 5 MOI, that is a multiplicity of infection (MOI) value of 5 reduced cell viability by approximately 50% in all BC cell lines. After 10 days, colonies were fixed with methanol and stained at room temperature for 30 min with crystal violet (Sigma-Aldrich, Merk KGaA, Darmstadt, Germany). Unless otherwise specified, ‘control’ refers to cells treated with vehicle only, without virus or drugs.

### 2.4 Protein extraction and western blot analysis

For total protein extraction, cells were lysed on ice for 30 min in lysis buffer containing 1 mM EDTA, 150 mM NaCl, 1%NP-40, 50 mM TRIS-HCL pH 7.5, and 10 mg/ml each of aprotinin, leupeptin, and pepstatin. Equal amounts of proteins (40μg) per sample were subjected to SDS-PAGE. Western blots were performed using antibodies against PARP (9542S, Cell Signaling, Technology, Danvers, Massachusetts, USA), pRB2/p130 (610262, BD Bioscience, San Jose, CA, USA), and GAPDH (2118S, Cell Signaling Technology, Danvers, Massachusetts, USA). Signals were detected using ECL (Amersham Biosciences).

### 2.5 Cytofluorimetric analysis of cell cycle profile

For cell cycle analysis, all cell lines were infected with CpHV-1 at 5 MOI, and 48h p.i., cells were collected, washed with PBS, and then fixed in 70% ice-cold ethanol. The cells were then incubated at 37°C for 1 h with 50 μg/ml propidium iodide (PI; Cat. no. P4170; Sigma-Aldrich, Merk KGaA, Darmstadt, Germany) and 20 μg/ml RNase (Cat. no. 9001 99 4; Sigma-Aldrich, Merk KGaA, Darmstadt, Germany) and then analyzed with BD FACSAria™ III and by the BD FACSDiva Software (BD Biosciences, San Jose, CA, USA).

### 2.6 Detection of apoptosis

For apoptosis detection, MCF-7, SKBR3 and MDA-MB-468 BC cell lines were seeded in duplicates in 96-well plates at a density of 2500 cells/well and allowed to adhere for 24h. We treated BC cells for 48h with CpHV-1 alone and with Abemaciclib (HR+ BC), Tucatinib (HER2+ BC), and Inavolisib (triple-negative BC) either alone or in combination at their respective IC50 values. At the same time, the RealTime-Glo™ Annexin V Apoptosis reagents were added to the medium following the manufacturer’s instructions (RealTime-Glo™ Annexin V Apoptosis and Necrosis Assay, Promega). Luminescence signals were measured using the VictorX2 Multilabel Plate Reader (PerkinElmer).

### 2.7 Drug combination studies

We treated the estrogen receptor-positive MCF-7 BC cells with CpHv-1 and Abemaciclib (A) alone or in combination, the HER2-positive SKBR3 BC cells with CpHV-1 and Tucatinib (T) alone or in combination, and the triple-negative MDA-MB-468 cells with CpHV-1 and Inavolisib (I). All cells were treated for 72h at various concentrations of the tested drugs in a constant ratio before being assessed for cell viability using the MTS assay. Synergism, additivity, or antagonism were determined using the Chou–Talalay method implemented in Compusyn software 1.0 (ComboSyn, Inc., Paramus, NJ 07652, USA). According to this approach, the Combination Index (CI) is calculated based on the median-effect principle: CI < 1 indicates synergism, CI = 1 indicates additivity, and CI > 1 indicates antagonism. The correlation coefficient (r value) reflects the goodness of fit of the data to the median-effect plot, with values close to 1 indicating high reliability of the CI calculation.

### 2.8 Statistical analysis

Statistical analyses were conducted using GraphPad Prism (version 10 for Windows). To compare all groups against the control group, a one-way repeated measures ANOVA followed by Dunnett’s *post hoc* test was applied. For multiple comparisons Tukey or Bonferroni post-tests were used. A p-value <0.05 was considered statistically significant.

## 3 Results

### 3.1 CpHV-1 reduces BC cell viability and clonogenic potential

**Figure 1:**
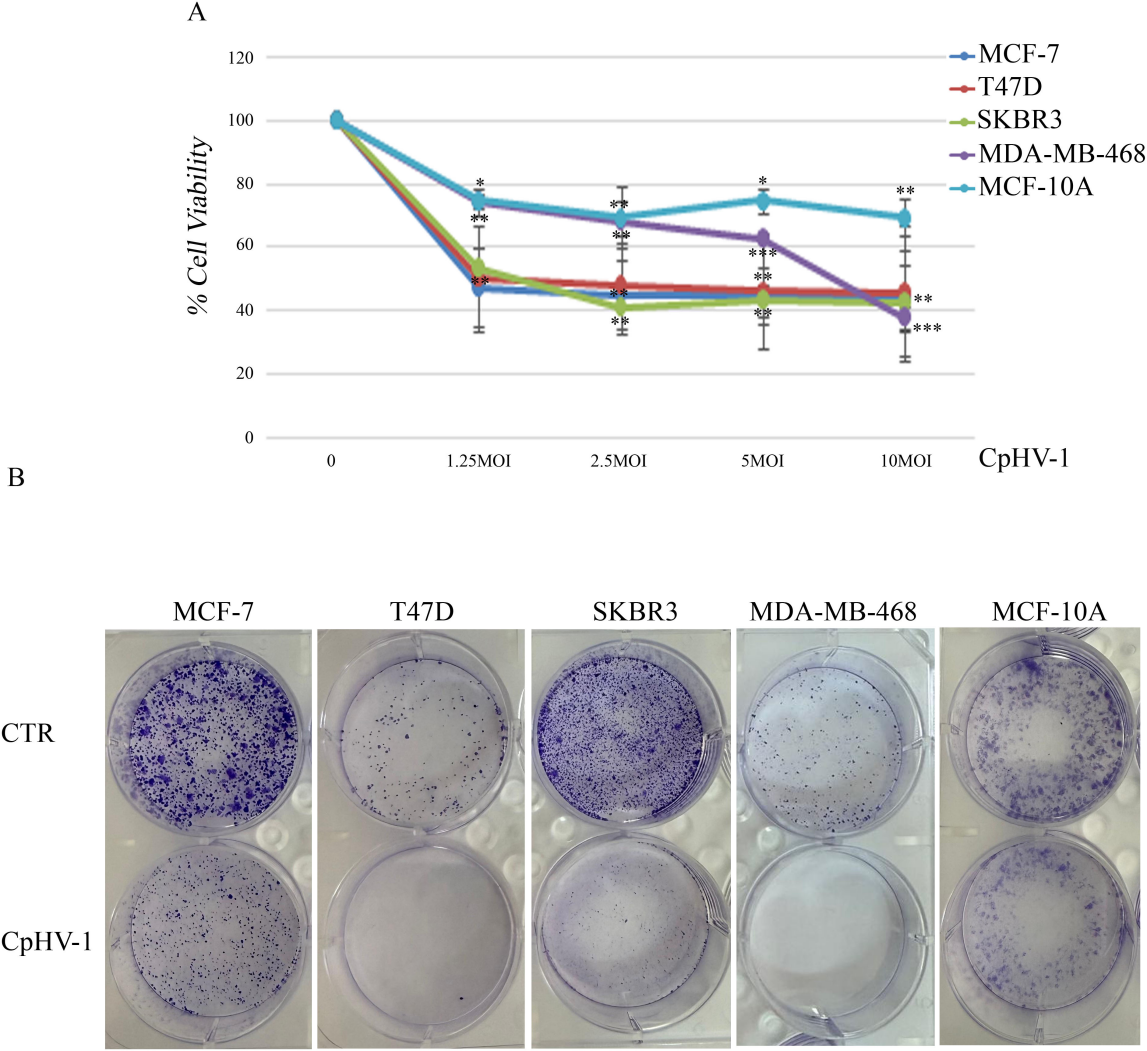
Effect of caprine herpesvirus 1 (CpHV-1) on breast cancer cell viability and clonogenic potential. **(A)** Dose–response curves reporting the effects of four different MOI (multiplicity of infection) of CpHV-1 on cell viability, evaluated through MTS assay at 72h post-infection. This assay was performed in four BC cell lines (MCF-7, T47D, SKBR3 and MDA-MB-468) and non-tumorigenic mammary epithelial cells (MCF-10A). The results are reported as the means ± standard deviation of at least three independent experiments, each conducted in triplicate, and expressed as percentages of cell viability (normalized to the untreated control cells). Statistically significant differences were evaluated by Anova with Dunnet post-test and indicated with: *significant (P < 0.05); **very significant (P < 0.01); and ***extremely significant (P < 0.001). **(B)** Long-term CpHV-1 effects were assessed by clonogenic assay. Colonies were stained with crystal violet 10 days after a 72h treatment with CpHV-1 (5 MOI). Representative plates from two independent experiments are shown.

We first assessed, using the MTS assay, the effect of CpHV-1 on the viability of BC cells (MCF-7, T47D, SKBR3, and MDA-MB-468) and non-tumorigenic mammary epithelial cells (MCF10-A) at 72 h after treatment (**Figure 1A**). The infection exhibited a time- and dose-dependent cytotoxic effect in all BC cell lines, without significantly affecting normal cells. Interestingly, CpHV-1 induced a cytotoxic effect also in the most aggressive triple-negative (TNBC) histotype (**Supplementary Figure S1**). In particular, we observed that a multiplicity of infection (MOI) value of 5 reduced cell viability by approximately 50% in all BC cell lines.

To verify whether CpHV-1 exerted long-term cell growth inhibition, we performed clonogenic assays upon infection with 5 MOI of CpHV-1. We found that the OV significantly reduced colony formation in all BC cell lines (**Figure 1B**).

### 3.2 CpHV-1 induces apoptosis in BC cells

**Figure 2:**
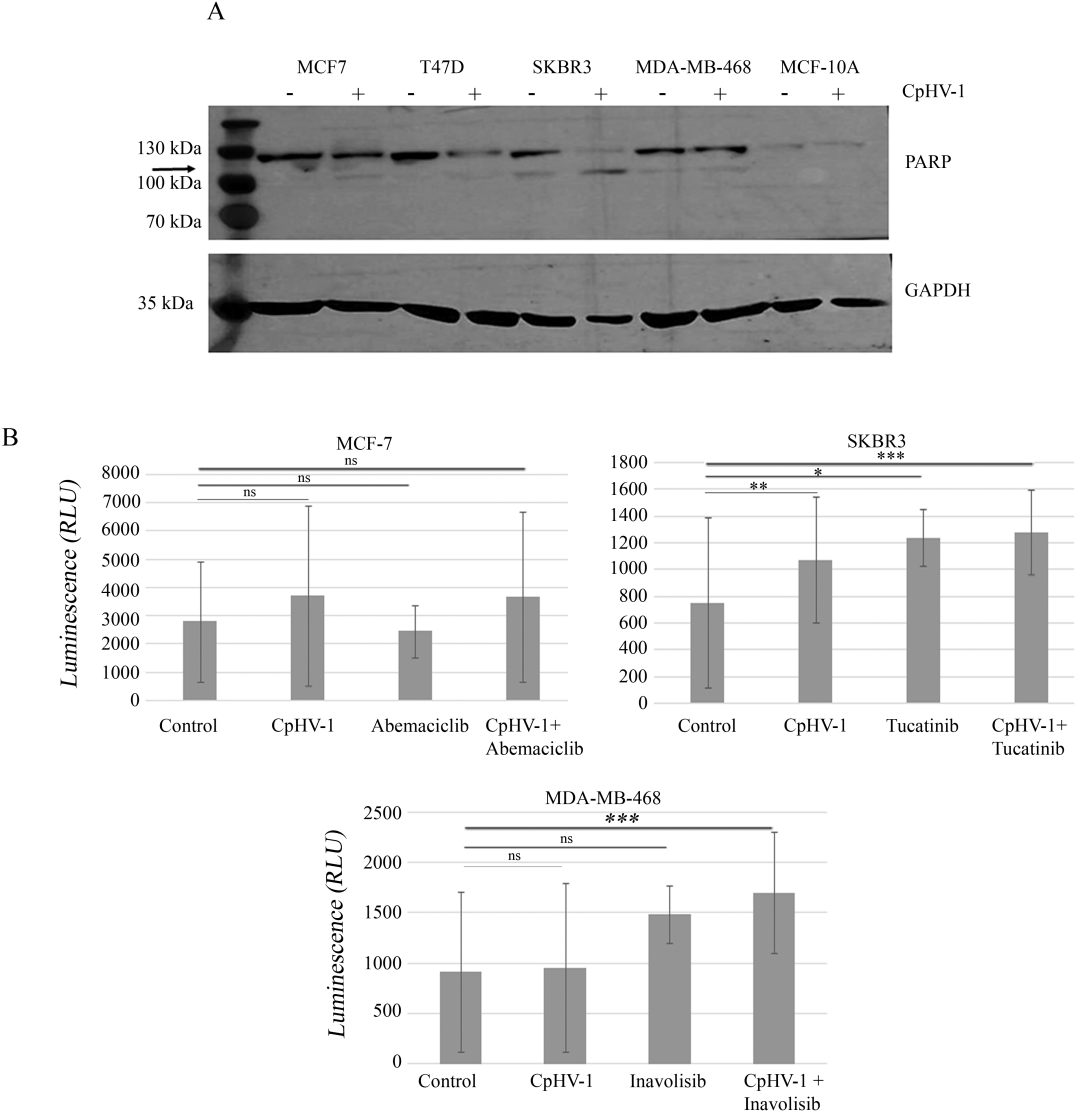
Apoptosis induction in breast cancer cells infected with caprine herpesvirus 1 (CpHV-1). **(A)** Western blot analysis of poly (ADP-ribose) polymerase (PARP) on MCF-7, T47D, SKBR3, MDA-MB-468 and MCF-10A treated for 72h with 5 MOI of CpHV-1. The anti-PARP antibody detects both the full-length proteins and the active, cleaved form. An anti-GAPDH antibody was used for loading control. The arrow indicates cleaved form of PARP **(B)** The graphs represent apoptosis evaluation 48 hours post-infection (p.i.) with 5 MOI of CpHV-1, abemaciclib, or their combination in MCF-7 cells; SKBR3 cells treated with CpHV-1, tucatinib, or their combination; and MDA-MB-468 cells treated with CpHV-1, inavolisib, or their combination. Apoptosis was assessed using the RealTime-Glo™ Annexin V Apoptosis Assay (Promega), which detects phosphatidylserine exposure through luminescent signal generation in MCF-7, SKBR3, and MDA-MB-468 cells. The control condition corresponds to cells treated with vehicle only (without virus or drugs). Luminescence intensity reflects the degree of apoptosis induced by CpHV-1 and the different treatments in each cell line. Statistically significant differences were evaluated by ANOVA with Dunnett’s post-test and indicated as follows: *P < 0.05; **P < 0.01; ***P < 0.001, ns, not statistically significant relative to control. The Annexin V apoptosis assay was performed in almost two independent experiments, each including two biological replicates.

To characterize the mechanism underlying CpHV-1 effects on BC cell viability, we assessed apoptosis induction by CpHV-1 in all cell lines. First, we evaluated the activation of the apoptotic marker poly (ADP-ribose) polymerase (PARP) by Western blot analysis 48 h post-infection (p.i.). Our data showed that CpHV-1 treatment caused a decrease in the full-length proteins and a concomitant increase in their active cleaved forms in BC cells but not in MCF-10A (**Figure 2A**). We then analyzed another apoptotic marker, NOXA, using qRT-Real Time PCR. Our results showed that NOXA expression was increased in all BC-treated samples compared to the control; conversely, no such increase was observed in normal cells (data not shown). To further confirm these observations, we employed the RealTime-Glo™ Annexin V Apoptosis and Necrosis Assay to monitor phosphatidylserine (PS) exposure on the outer leaflet of the plasma membrane. A luminescence signal was detected in the three BC cell lines tested (MCF-7, SKBR3, and MDA-MB-468) (**Figure 2B**). For MCF-7 cells, treatments included CpHV-1 in combination with abemaciclib, as well as the single agents (CpHV-1 alone or abemaciclib alone). For SKBR3 cells, we evaluated tucatinib both alone and in combination with CpHV-1, and for MDA-MB-468 cells, we tested inavolisib as a single agent and in combination with CpHV-1. The Annexin V positivity observed in these settings was consistent with our previous study in MDA-MB-468 cells (21). Altogether, these preliminary results indicate that CpHV-1 treatment is associated with modulation of apoptosis–related markers across BC subtypes and suggest a selective effect when combined with clinically relevant targeted therapies, highlighting its potential therapeutic applicability irrespective of molecular classification.

### 3.3 CpHV-1 perturbs BC cell cycle progression

**Figure 3:**
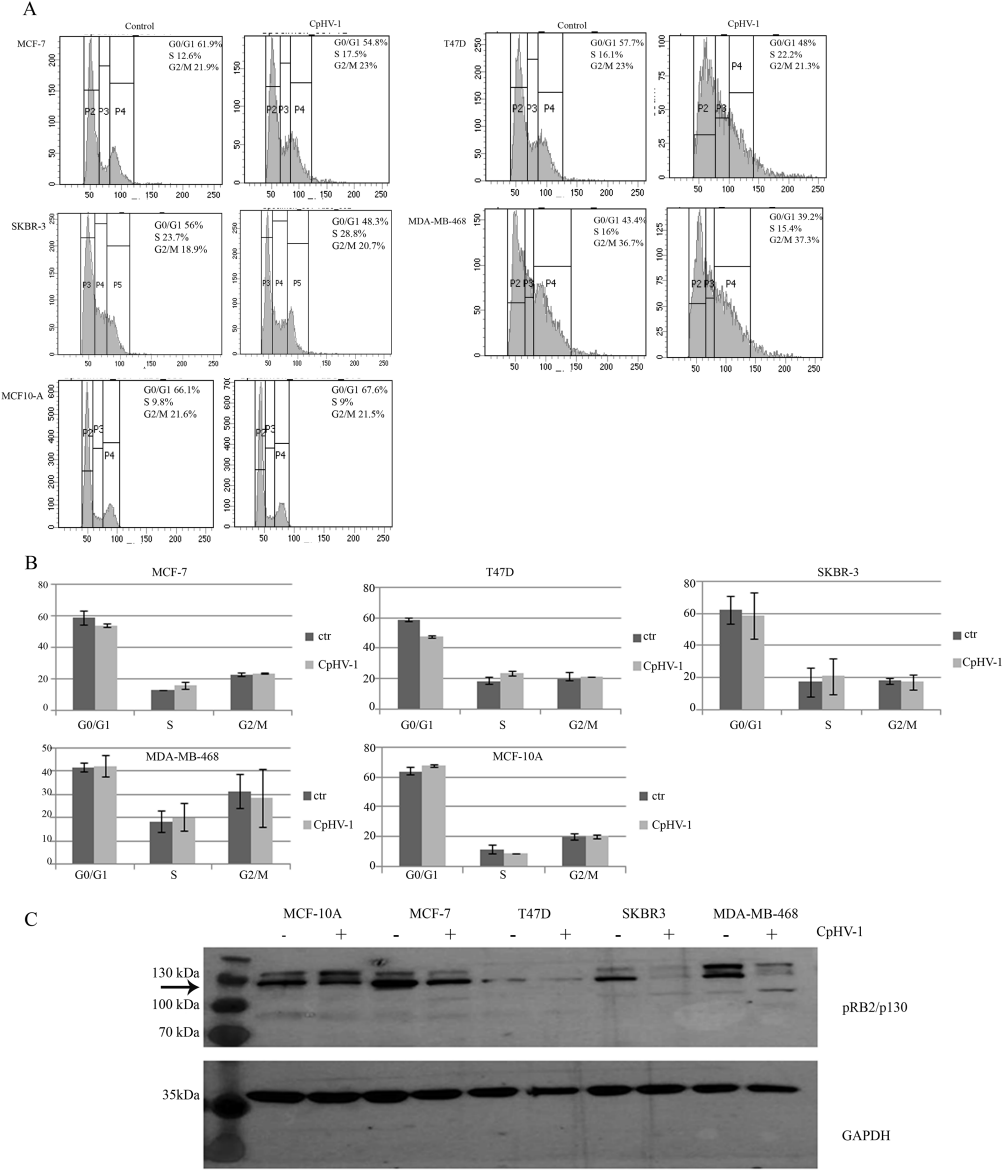
Effect of caprine herpesvirus 1 (CpHV-1) on breast cancer cell cycle progression. **(A)** Representative cell cycle profile, out of 2 independent FACS analyses performed on each cell line (MCF-7, T47D, SKBR3, MDA-MB-468 and MCF-10A) upon 48 p.i with CpHV-1 (5 MOI) is shown. **(B)** The graphs represent the mean ± standard deviation (SD) of two independent experiments showing the different phases of the cell cycle and their modulation following CpHV-1 infection in BC cancer cells and in normal MCF-10Acells. Treatment times and doses are the same as previously indicated. **(C)** Western blot analysis of pRB2/p130 on MCF-7, T47D, SKBR3, MDA-MB-231 and MDA-MB-468 treated for 48h with 5 MOI of CpHV-1. An anti-GAPDH antibody was used for loading control. The arrow indicates RBL2/p130-specific band (26).

We assessed the effects of CpHV-1 infection on BC cell cycle progression by evaluating cellular DNA content using FACS analysis 48h p.i. We found that CpHV-1 affected the distribution of BC cell cycle phases. In particular, we observed an increase in the S-phase cell population (**Figure 3A**). To better visualize these differences, the quantitative distribution of cells across the various cell cycle phases is shown in the corresponding bar graph (**Figure 3B**), which highlights the enrichment of S-phase cells following CpHV-1 infection. We also analyzed the protein expression levels of the pRB2/p130, a key regulator involved in cell cycle exit and maintenance of the G0/G1 phase. Western blot analysis revealed a consistent decrease in pRB2/p130 levels across all BC cell lines infected with 5 MOI of CpHV-1 (**Figure 3C**). This downregulation was observed 48 hours p.i. and aligns with the expected role of OVs in disrupting cell cycle progression to favor viral replication. The modulation of pRB2/p130 levels could reflect a mechanism employed by the virus to hijack host cell machinery and may contribute to the observed oncolytic activity. This disruption facilitates the transition from quiescence to active cycling, promoting S-phase entry and enhancing the cellular environment for viral replication.

### 3.4 CpHV-1 synergizes with Abemaciclib, Tucaticinb and Inavolisib in suppressing BC cell viability

**Figure 4:**
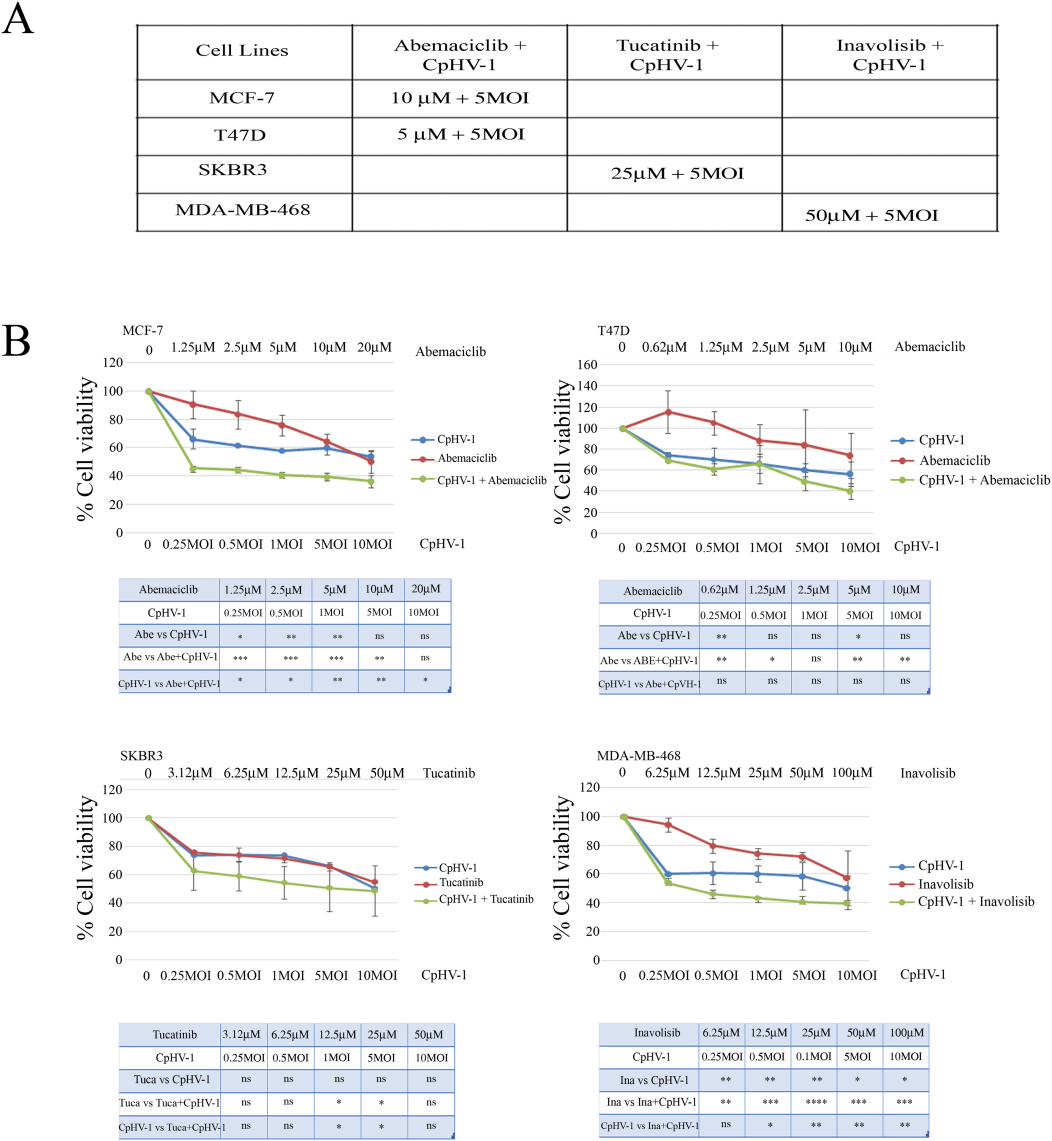
Synergistic effect of caprine herpesvirus 1 (CpHV-1) and abemaciclib, tucatinib and inavolisib on breast cancer cell lines. **(A)** The table reports the IC50 values at 72h of Abemaciclib, Tucatinib and Inavolisib on BC cell lines, as determined through MTS analysis of cell viability. **(B)** Dose-response curves of CpHV-1 alone, and abemaciclib, tucatinib and inavolisib alone, and both agents in combination in MCF-7, T47D, SKBR3, MDA-MB-468 cell lines 72h after treatment. Results represent the means ± standard deviation of 2 independent experiments, each conducted in triplicate, and are expressed as percentages of cell viability calculated with respect to control cells treated with DMSO alone. In the lower panel significant differences, obtained from one-way ANOVA with Tukey’s post-test to compare single treatment versus OVs, and single agents versus combination are indicated with *P-value < 0.05,**P-value<0.01,***P-value<0.001, ****Pvalue< 0.0001, ns not statistically significant.

**Figure 5:**
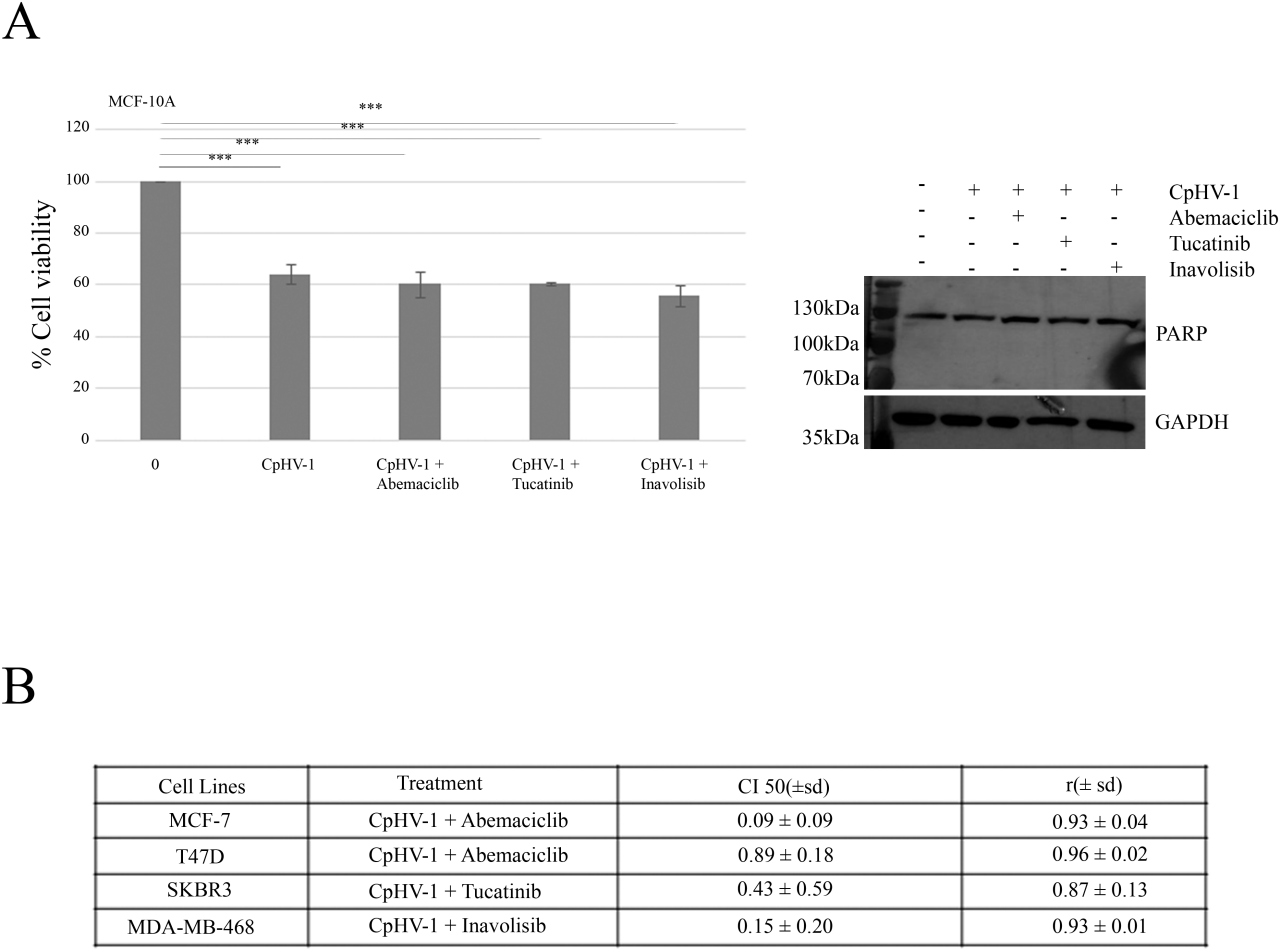
Interactions between CpHV-1 and targeted therapies in MCF-10A non-neoplastic cells and BC cells **(A)** Left: Histogram showing that 72 h of treatment with CpHV-1 and three combinations at the indicated doses had no significant toxic effect on the normal cells MCF-10A, as assessed through MTS and evaluated by one-way repeated measures ANOVA with Dunnet post-test to compare combination versus control are indicated with ***P-value<0.001. Results are reported as means ± standard deviations of two independent experiments and expressed as percentages of cell viability relative to control cells treated with DMSO alone. Right: Representative Western blot analysis showing total PARP, with no detection of cleaved PARP, in normal breast epithelial MCF10A cells treated with CpHV-1 alone or in combination with abemaciclib, tucatinib, or inavolisib. The absence of cleaved PARP indicates that apoptosis does not occur in this cell line. GAPDH was used as a loading control. **(B)** Table reporting the means ± standard deviations of combination index (CI) and r values of the CpHV-1 and three combinations at 50% of cell killing (CI50) following 72h of treatment, calculated by the Compusyn software for each of the 2 independent experiments. Consistent CI values < 1 were obtained across all breast cancer subtypes tested, supporting a synergistic interaction between CpHV-1 and the targeted drugs. The corresponding r values were close to 1, confirming the robustness and reliability of the CI calculations.

We next examined the possible synergistic effects of CpHV-1 in combination with Abemaciclib (A), Tucatinib (T), and Inavolisib (I) using the MTS assay. MCF-7, T47D, SKBR3, and MDA-MB-468 cells were treated for 72 h with CpHV-1 alone, each drug alone, or their combinations at various concentrations in a constant ratio. Specifically, A was tested in MCF-7 and T47D cells, whereas T and I were tested in SKBR3 and MDA-MB-468 cells, respectively. The agents were applied in serial dilutions above and below 5 MOI of CpHV-1 at multiple concentrations above and below the IC50 of each agent (**Figure 4A**, Suppl.2). The specific concentrations used and the corresponding percentages of cell viability are reported in **Figure 4B**. Combination index (CI) analysis using the Chou–Talalay method revealed synergistic interactions (CI < 1) for all tested cell lines (**Figure 5B**). To exclude potential cytotoxic effects of these combinations on non-neoplastic cells, normal MCF-10A cells were exposed to three CpHV-1–drug combinations. No significant toxic effects were observed after 72 h of treatment (**Figure 5A**). The reduction (~40%) in viability observed at the highest viral doses likely reflects experimental forcing of the system, since under the same conditions BC cells showed a marked 70–80% reduction in viability. This confirms the relative resistance of normal cells to CpHV-1. Finally, Western blot analysis of PARP expression, a marker of apoptosis, showed no significant differences between untreated MCF-10A cells and those exposed to the CpHV-1 combinations, further supporting the absence of cytotoxicity in non-neoplastic cells (**Figure 5A**).

## 4 Discussion

BC remains a leading cause of cancer-related mortality worldwide (10). Despite the progress made in understanding the molecular mechanisms driving BC and the development of effective therapy, resistance and toxicity make metastatic BC a still lethal disease (27). Therefore, new therapeutic strategies are urgently needed that can enhance treatment efficacy while minimizing toxicity.

OVT has gained attention as a promising therapeutic strategy for cancer treatment, with numerous clinical studies demonstrating its efficacy and safety (28). OVs exhibit significant antitumor activity, either as monotherapies or in combination with other therapeutic agents, offering a potentially effective treatment option for cancers, including BC (29, 30). Recent studies highlight the potential of OVs to selectively target tumor cells, exploiting molecular defects unique to cancer cells, and avoiding harm to normal tissues (31).

In this study, we investigated the effects of CpHV-1, a non-human, wild-type Alphaherpesvirinae subfamily virus, on a panel of BC cell lines and normal mammary epithelial cells (MCF-10A). Our results demonstrated that CpHV-1 significantly reduced the viability and clonogenic potential of all BC cell lines tested. Importantly, CpHV-1 exhibited no cytotoxic effects on normal mammary epithelial cells, confirming its selective oncolytic activity.

These findings are consistent with previous studies highlighting the selective nature of OVs (17, 32). The selective cytotoxicity of CpHV-1 observed in this study may be attributed to its ability to induce apoptosis in BC cells, as evidenced by the activation of key apoptotic markers, including Poly(ADP-ribose) polymerase (PARP) and the NOXA gene. These findings align with previous research indicating that CpH V-1 infection triggers apoptotic pathways specifically in malignant cells (7, 21). Most preclinical studies on OVs have primarily focused on TNBC, due to its aggressive nature and limited treatment options (30, 33). However, BC is a highly heterogeneous disease, comprising various molecular subtypes and this molecular diversity significantly influences tumor progression, therapeutic response, and clinical outcomes. Consequently, developing an OV that is effective across all subtypes remains a major challenge, as each subtype may display distinct susceptibilities to viral infection. In this study, the CpHV-1-based OV demonstrated consistent efficacy across all tested BC subtypes. This broad-spectrum activity highlights its potential as a versatile therapeutic agent capable of overcoming the limitations posed by BC heterogeneity. In addition to apoptosis, CpHV-1 infection was shown to modulate cell cycle progression in BC cells significantly. Western blot analysis revealed a decrease in the levels of pRb2/p130, a key regulator of the G0/G1 phase transition in the cell cycle. This observation suggests that CpHV-1 may target cell cycle control mechanisms, further contributing to its cytotoxic effect on cancer cells. These results are consistent with prior studies demonstrating that OVs can disrupt cell cycle regulation, potentially enhancing the therapeutic efficacy of oncolytic virotherapy (7, 21).

Finally, combining OVs with conventional or targeted therapies has emerged as a particularly promising strategy for BC treatment (22, 34). Here, we explored the synergistic effects of CpHV-1 in combination with targeted therapies for different BC subtypes. Specifically, we assessed the effects of CpHV-1 in combination with abemaciclib in HR+ BC cell lines, tucatinib in HER2+ BC cells, and inavolisib in triple-negative BC cells. Our results revealed that CpHV-1 exhibited synergy with all three agents, enhancing the sensitivity of BC cells to treatment without inducing significant toxicity in normal cells. These findings suggest that CpHV-1 could be used to sensitize BC cells to existing biological therapies, potentially improving treatment outcomes.

A limitation of the present study is the absence of a CpHV-1 variant that is replication-deficient or otherwise non-oncolytic as an internal viral negative control. The availability of such a control would permit more rigorous demonstration that observed cytotoxicity and pro-apoptotic effects are strictly dependent on active virus replication or specific viral functions. While similar approaches using attenuated or mutant viral strains have been adopted in oncolytic virus research (e.g. ICP34.5-deleted HSV variants, or mutants of polymerase genes) to rule out non-specific effects (35), such a variant is not currently available for CpHV-1 in our hands. Thus, we cannot formally exclude that some observed effects may include contributions from non-replicative viral components or viral entry processes. Future work should ideally include the engineering of a CpHV-1 mutant incapable of replication or a neutral viral backbone to serve as a control, to strengthen causality attribution.

Currently, Ovs tested for BC are predominantly armed or used in combination with other treatments to enhance their therapeutic effects (17). Adenoviruses are the most studied OVs in BC, particularly in TNBC, with modified versions like CNHK600-IL24 showing strong antitumor effects in combination settings (36). Other OVs such as G47D (HSV-based) and VG9-IL-24 (a recombinant vaccinia virus) also demonstrated significant cytotoxicity in BC models, again when used as combined therapeutic strategies (37). A recent case study involving CHECKvacc, a CF33-based poxvirus, demonstrated significant tumor regression and extended disease-free survival in a patient with metastatic triple-negative breast cancer (TNBC). Notably, these positive outcomes were achieved only after subsequent treatment with T-DXd, underscoring the critical importance of combining innovative immunotherapies with targeted treatments (38).

This evidence highlights the potential of combination or sequential approaches to improve outcomes in challenging cancer cases. Our study with CpHV-1 demonstrates that it may not require modification to be effective, as it shows potential for synergy with targeted therapies.

In conclusion, our findings support the targeted approach, demonstrating that CpHV-1 has a favorable therapeutic index, making it a promising candidate for clinical use in BC treatment. However, further research is needed to understand the mechanisms behind CpHV-1’s effectiveness in BC models and to assess its potential in both preclinical and clinical settings. The strategy tested in this study reduces the need for extensive viral modifications and provides a practical method for combining OVT with other treatments to enhance outcomes in BC.

## Data Availability

The datasets presented in this study can be found in online repositories. The names of the repository/repositories and accession number(s) can be found below: https://doi.org/10.5281/zenodo.16603322.

## Supplementary material

Supplementary Figure 1 Morphological and number changes of breast cancer cells after CPHV1 infection. The treatment induces modifications in all BC cell characteristics and number. The changes do not affect MCF-10A normal cells. Scale bar: 100 µm.

Supplementary Figure 2 Dose–response curves showing the effect of different inhibitors on breast cancer cell line viability. MCF-7 and 747D cells were treated with increasing concentrations of Abemaciclib, SKBR3 cells with Tucatinib and MDA-MB-468 cells with Inavolisib. Cell viability was assessed after treatment using MTS assay, and expressed as a percentage relative to untreated controls. The curves illustrate the reduction in cell viability in a dose-dependent manner, from which IC_50_ values were calculated.
