# Supplementary figures and images for: Caprine herpes virus-1 reduces cell viability and enhances chemosensitivity in breast cancer cells
Source: Front Oncol. 2025 Nov 24;15:1676296. doi: 10.3389/fonc.2025.1676296 (PMC12682693; doi:10.3389/fonc.2025.1676296)

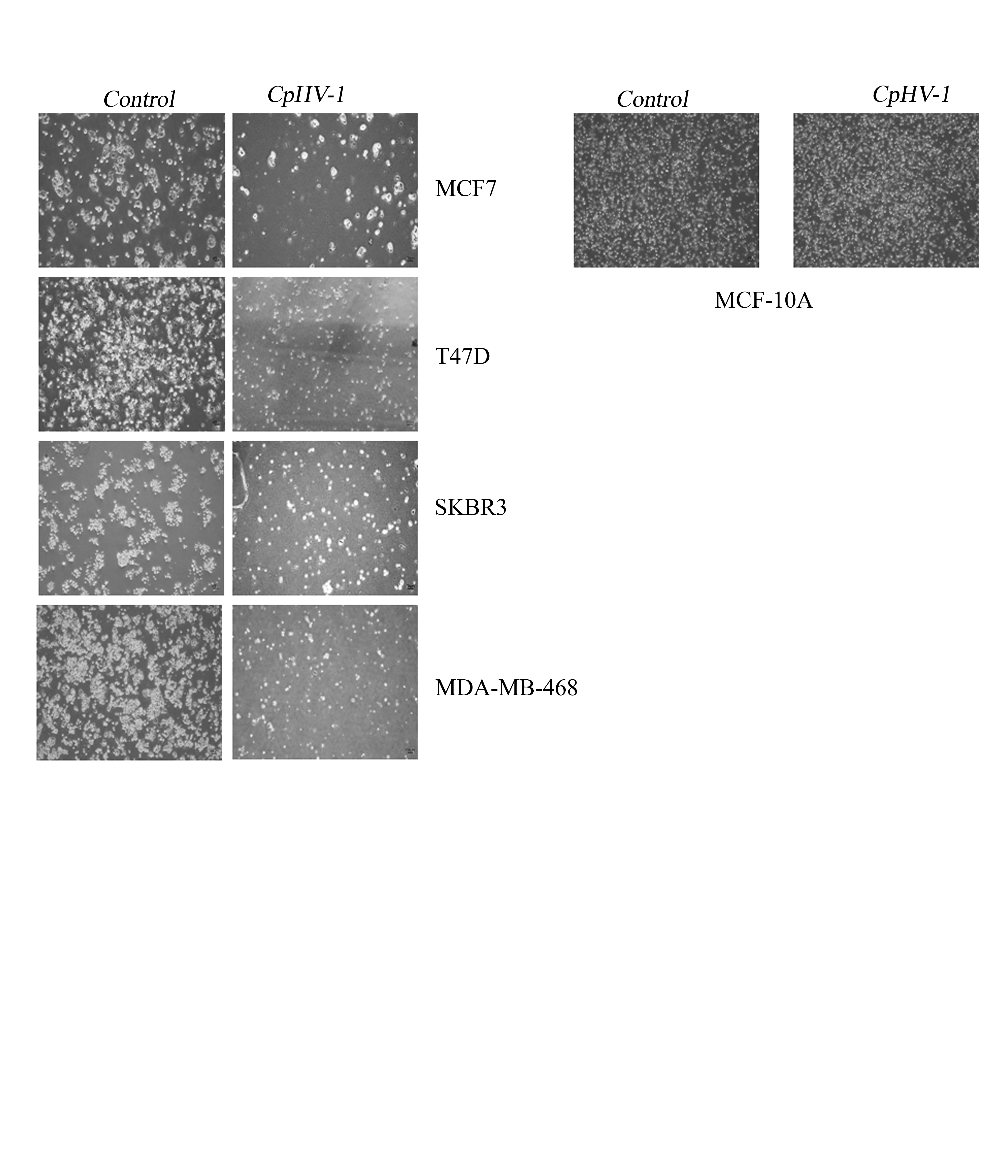

Supplement: Supplementary Figure 1 — Morphological and number changes of breast cancer cells after CPHV1 infection. The treatment induces modifications in all BC cell characteristics and number. The changes do not affect MCF-10A normal cells. Scale bar: 100 µm. [file DataSheet1.zip › SUPPLEMENTARY 1.tif]

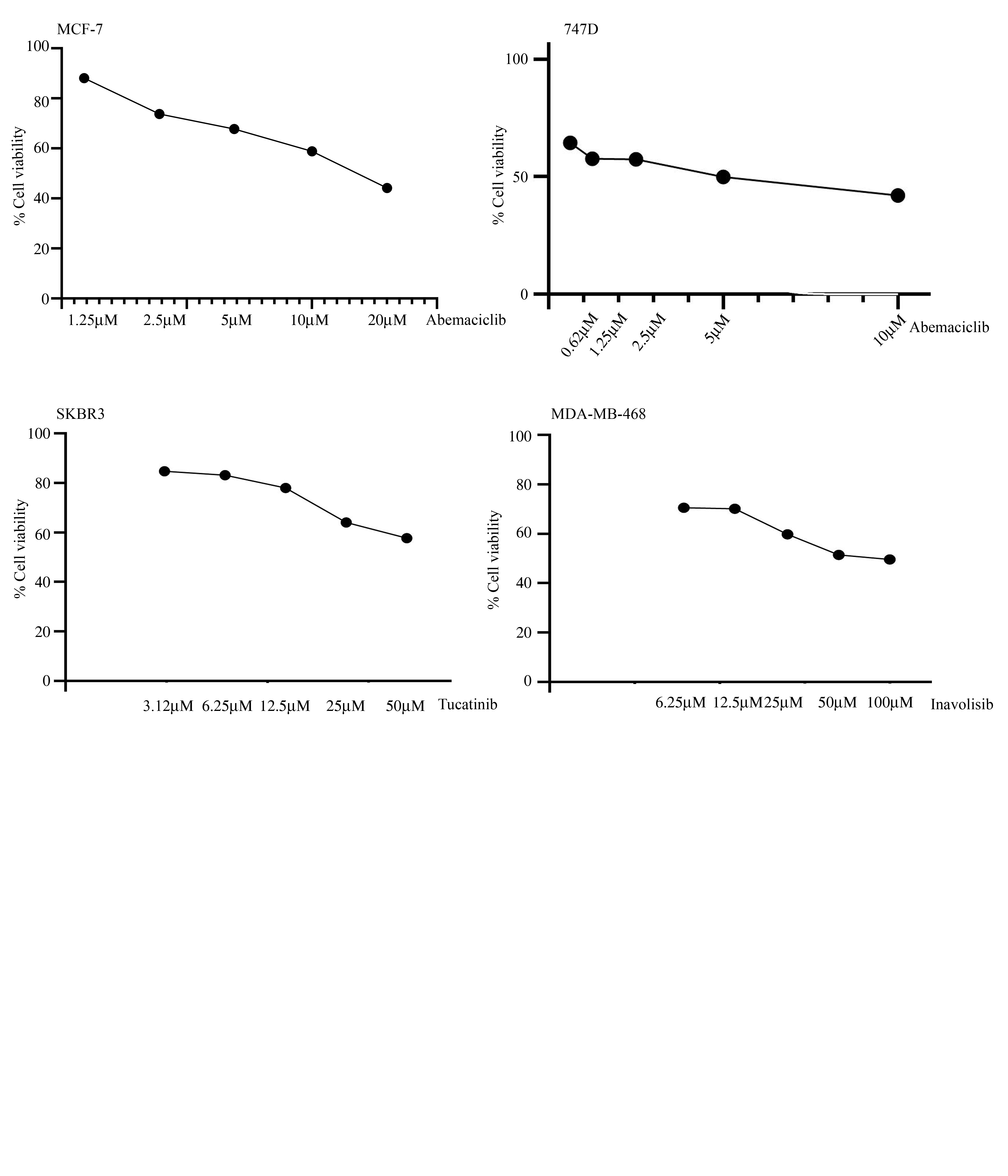

Supplement: Supplementary Figure 2 — Dose–response curves showing the effect of different inhibitors on breast cancer cell line viability. MCF-7 and 747D cells were treated with increasing concentrations of Abemaciclib, SKBR3 cells with Tucatinib and MDA-MB-468 cells with Inavolisib. Cell viability was assessed after treatment using MTS assay, and expressed as a percentage relative to untreated controls. The curves illustrate the reduction in cell viability in a dose-dependent manner, from which IC50 values were calculated. [file Image1.tif]
